# Supplementary figures and images for: A Novel Multi-Step Global Mechanism Scheme for n-Decane Combustion
Source: Entropy (Basel). 2023 Sep 28;25(10):1389. doi: 10.3390/e25101389 (PMC10606497; doi:10.3390/e25101389)

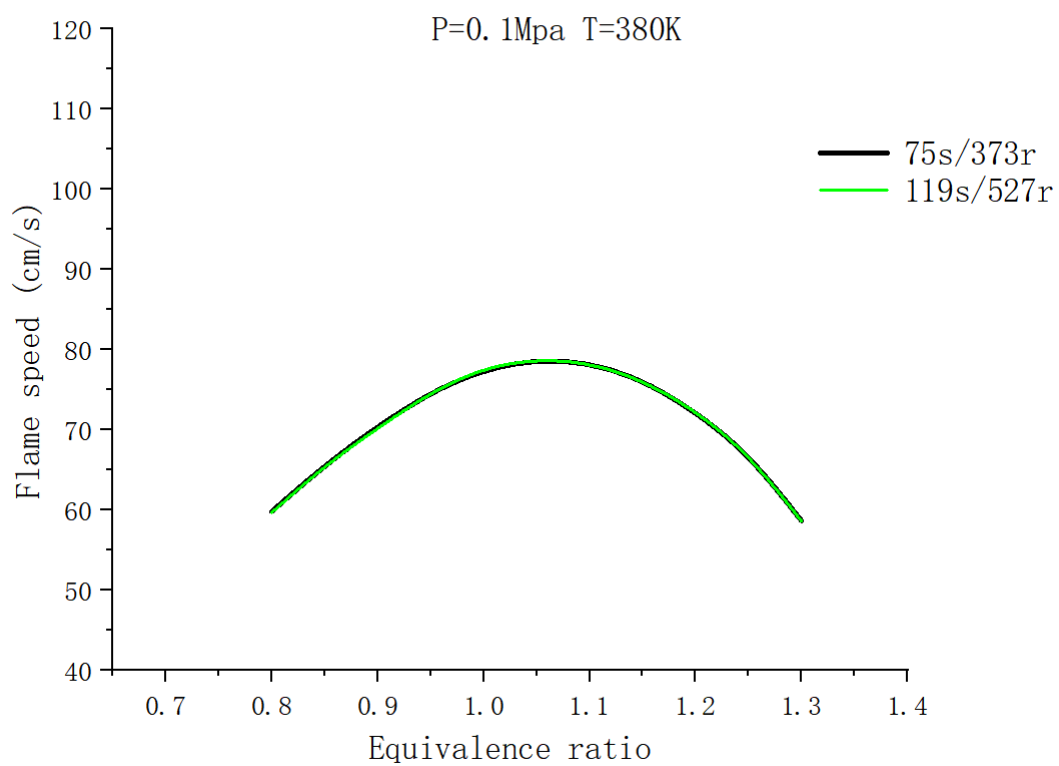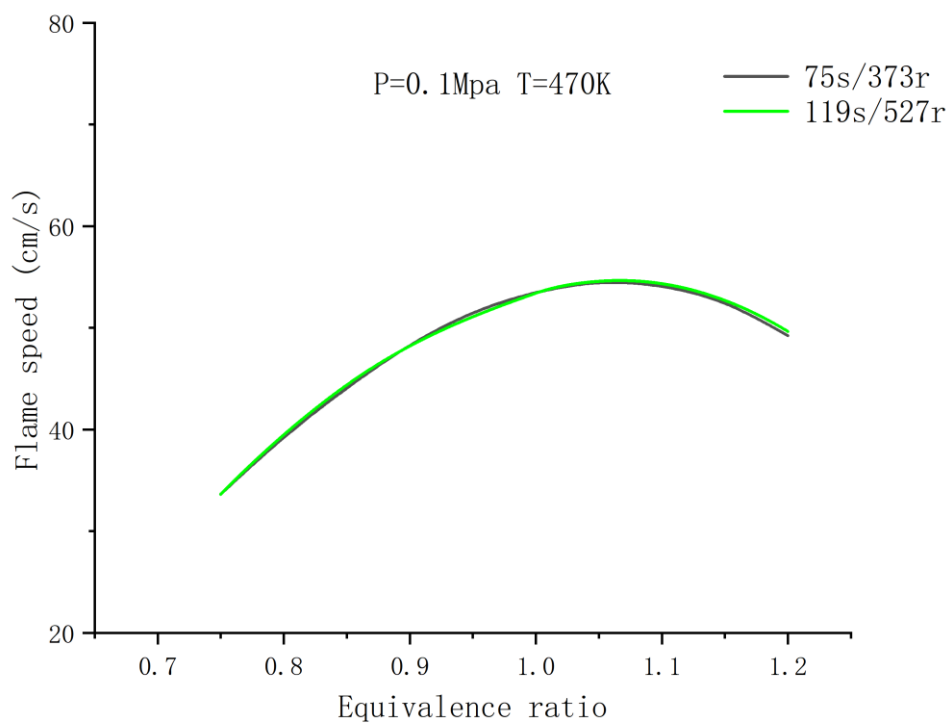

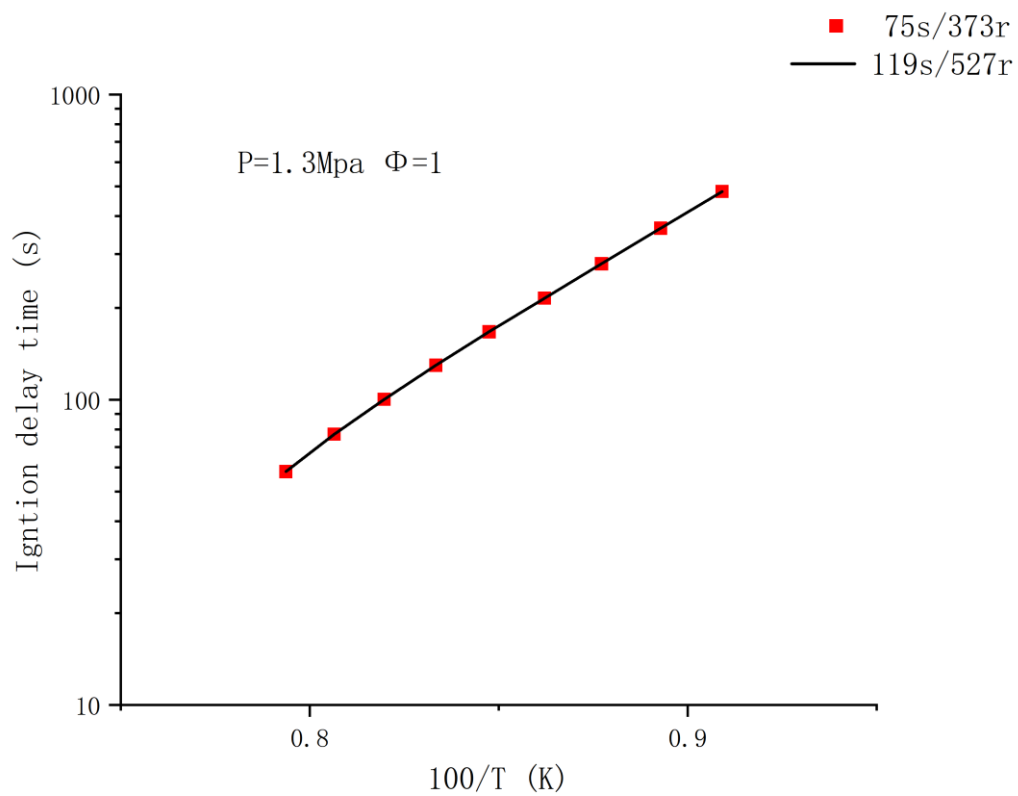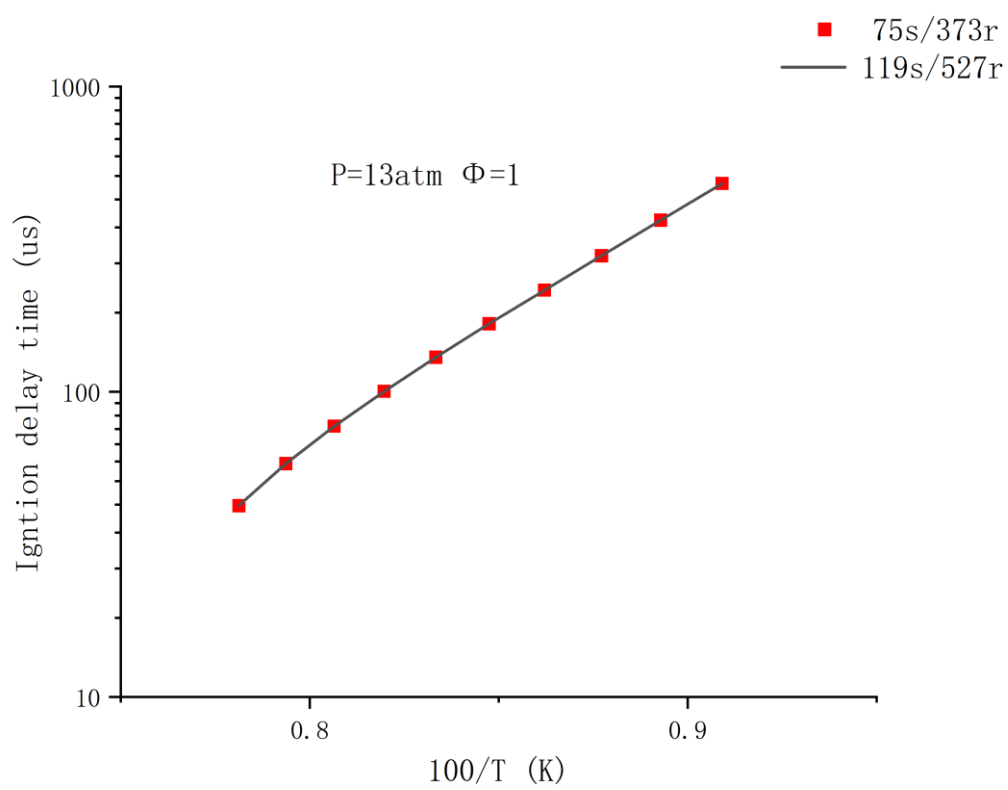

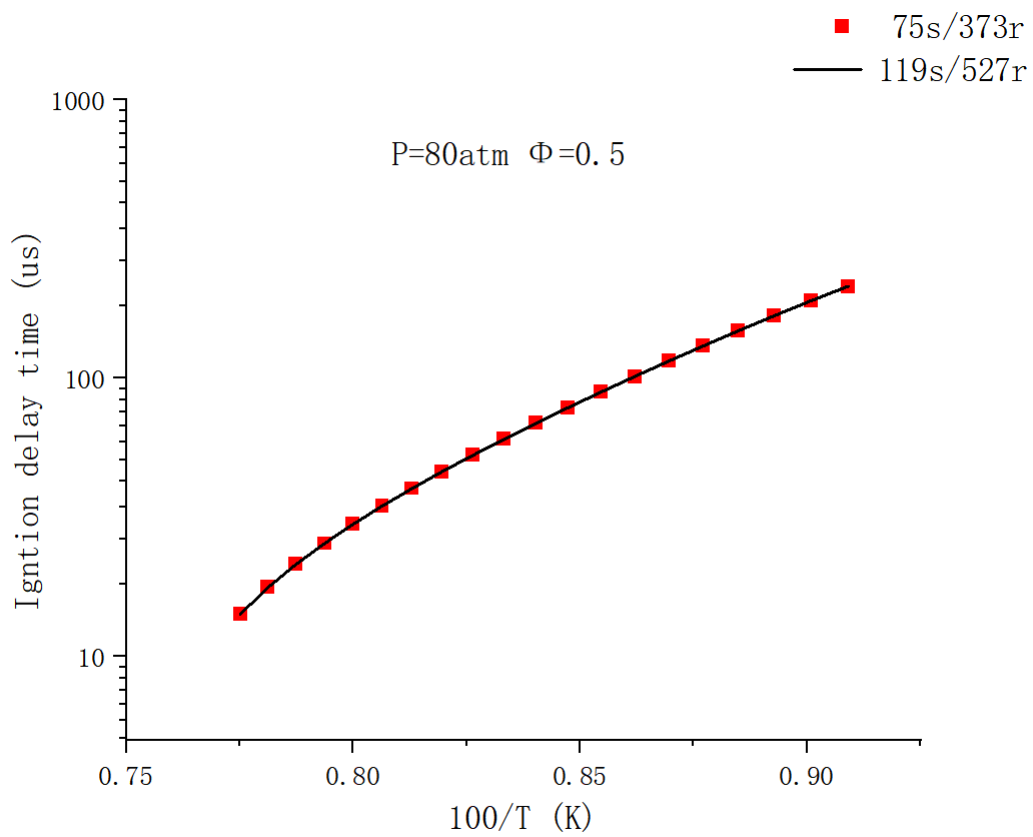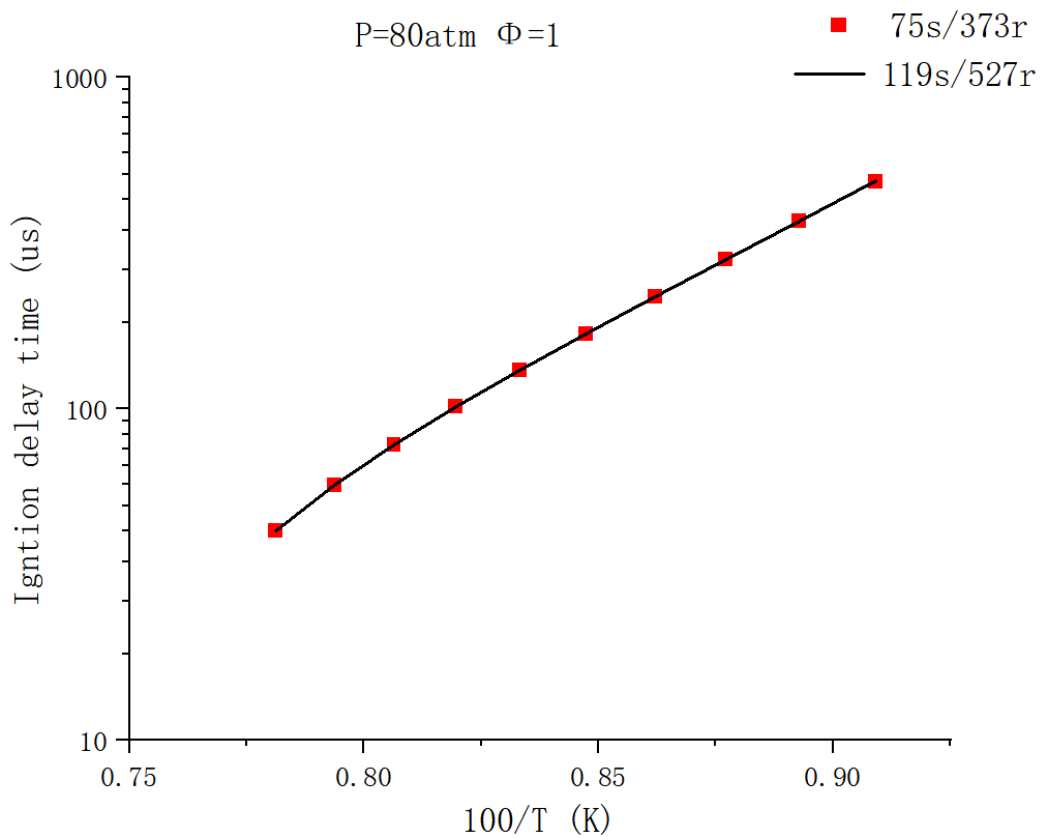

Supplement: Supplementary file 1 [file entropy-25-01389-s001.zip › supplementary material.pdf]
